# Exploring experimental cerebral malaria pathogenesis through the characterisation of host-derived plasma microparticle protein content

**DOI:** 10.1038/srep37871

**Published:** 2016-12-05

**Authors:** Natalia Tiberti, Sharissa L. Latham, Stephen Bush, Amy Cohen, Robert O. Opoka, Chandy C. John, Annette Juillard, Georges E. Grau, Valéry Combes

**Affiliations:** 1 School of Life Sciences, Faculty of Sciences, University of Technology Sydney, Australia; 2 Vascular Immunology Unit, Department of Pathology, The University of Sydney, Australia; 3 School of Mathematical and Physical Sciences, University of Technology Sydney, Australia; 4 College of Health Sciences, Makerere University, Kampala, Uganda; 5 Indiana University School of Medicine, Indianapolis, United States of America; 6 University of Minnesota, Minneapolis, United States of America; 7 La Jolla Infectious Diseases Institute, San Diego, CA, United States of America

## Abstract

Cerebral malaria (CM) is a severe complication of *Plasmodium falciparum* infection responsible for thousands of deaths in children in sub-Saharan Africa. CM pathogenesis remains incompletely understood but a number of effectors have been proposed, including plasma microparticles (MP). MP numbers are increased in CM patients’ circulation and, in the mouse model, they can be localised within inflamed vessels, suggesting their involvement in vascular damage. In the present work we define, for the first time, the protein cargo of MP during experimental cerebral malaria (ECM) with the overarching hypothesis that this characterisation could help understand CM pathogenesis. Using qualitative and quantitative high-throughput proteomics we compared MP proteins from non-infected and *P*. *berghei* ANKA-infected mice. More than 360 proteins were identified, 60 of which were differentially abundant, as determined by quantitative comparison using TMT^TM^ isobaric labelling. Network analyses showed that ECM MP carry proteins implicated in molecular mechanisms relevant to CM pathogenesis, including endothelial activation. Among these proteins, the strict association of carbonic anhydrase I and S100A8 with ECM was verified by western blot on MP from DBA/1 and C57BL/6 mice. These results demonstrate that MP protein cargo represents a novel ECM pathogenic trait to consider in the understanding of CM pathogenesis.

Malaria infection caused by *Plasmodium* protozoan parasites still represents a major worldwide health problem affecting more than 200 million people and being responsible for the death of 600,000 of them, according to the latest WHO estimations^1^. Cerebral malaria (CM) is the most fatal malaria complication and affects mainly children under the age of 5 in sub-Saharan Africa^2^. CM prompt diagnosis remains difficult^2^ and despite available treatment, 15–20% of patients die, while 10–15% of cured patients will suffer from long-term neurological deficits^3^.

The pathological mechanisms of this complex neurological syndrome are still to be fully deciphered. The best described processes of CM pathogenesis include the sequestration of parasitized red blood cells (pRBC) in the brain microvasculature and an excessive activation of the immune response with production of pro-inflammatory cytokines^4^. An additional important feature is the increased number of microparticles (MP) in patients’ circulation. MP are submicron extracellular vesicles (100–1000 nm in size) released through a mechanism of outward blebbing of the plasma membrane by potentially all host cell types under physiological conditions or following stress and apoptosis^5^. Due to their process of formation, MP expose on their surface negatively charged phosphatidylserine residues and cellular markers specific to their cell of origin^6^. Importantly, they contain proteins, nucleic acids and lipids derived from the cytoplasm of the parent cell that they can convey to target cells, thus playing an important role in the intercellular communication and exchange of biological information^7^ as already shown in cancers, neurological disorders and cardiovascular diseases^8^,^9^,^10^. Interestingly, the proteomics characterisation of the cargo of plasma MP released under specific pathological conditions has already been found useful to identify new disease biomarkers and to propose new pathophysiological features^11^,^12^.

In CM patients, an increased number of MP originating from platelets, endothelial cells, monocytes and red blood cells has been shown in different clinical cohorts, where a significant correlation with the disease severity was proven^13^,^14^,^15^. Studies in the murine model of CM (experimental cerebral malaria – ECM), consisting of susceptible mouse strains (DBA/1, CBA and C57BL/6) infected with the *Plasmodium berghei* ANKA (PbA) parasite^16^, significantly contributed to further demonstrating that MP are not merely inert cellular products but active players in CM progression^17^,^18^. Indeed, similarly to human CM, increased numbers of cell-specific MP have been detected in ECM^18^ and mice showing a reduced release of MP (ABCA1^−/−^ or pantethine-treated) are protected from the cerebral syndrome^17^,^19^. Additionally, adoptively transferred ECM plasma MP localised in inflamed vessels, elicited breakdown of the blood brain barrier and brain pathology similar to ECM^18^.

Although an important role of circulating MP in CM pathogenesis is now generally accepted^20^,^21^, the mechanisms through which these vesicles carry out their biological functions still need to be deciphered and their protein cargo is yet to be described. In this context, we hypothesized that the protein content of circulating host-derived MP produced during CM might be of central importance in the pathogenesis of this syndrome. To closely follow the development of the neurological syndrome, we took advantage of the well-established CM mouse model and we investigated the protein content of MP produced during ECM using high-throughput qualitative and quantitative proteomics and network analyses. Two selected ECM-MP proteins, carbonic anhydrase 1 (CA-I) and S100A8, were further verified on a larger number of samples and their abundance was proven to be increased within plasma MP specifically released during the infection.

## Results

### Experimental design

To obtain quantitative information on the protein cargo of MP released during different infection conditions we used the Tandem Mass Tag^TM^ (TMT) isobaric labelling technology (ThermoFisher Scientific).

To set up an efficient quantitative protocol to analyse plasma MP proteins obtained from individual mice, the TMTzero (TMT^0^) labelling approach was first applied. MP from one non-infected (NI) and one PbA-infected mouse (experimental cerebral malaria – ECM) were used. The lists of identified proteins were considered as “qualitative datasets”.

The protocol established with the TMT^0^ was then translated to the TMTsixplex (TMT^6^) format. To increase the number of tested samples, two parallel experiments were performed comparing plasma MP proteins from non-infected mice (NI, n = 4), PbA-infected mice at day 3 post-infection (d3 pi, n = 4) and PbA-infected mice at d8 post-infection when all the signs of cerebral malaria are detected (ECM, n = 4).

Qualitative and quantitative proteomics results were then compared and evaluated in the context of CM pathogenesis through gene ontology and network analyses, and further verified by western blot (WB) for two selected proteins, carbonic anhydrase I (CA-I) and S100A8 on two different mouse strains.

**Figure 1:**
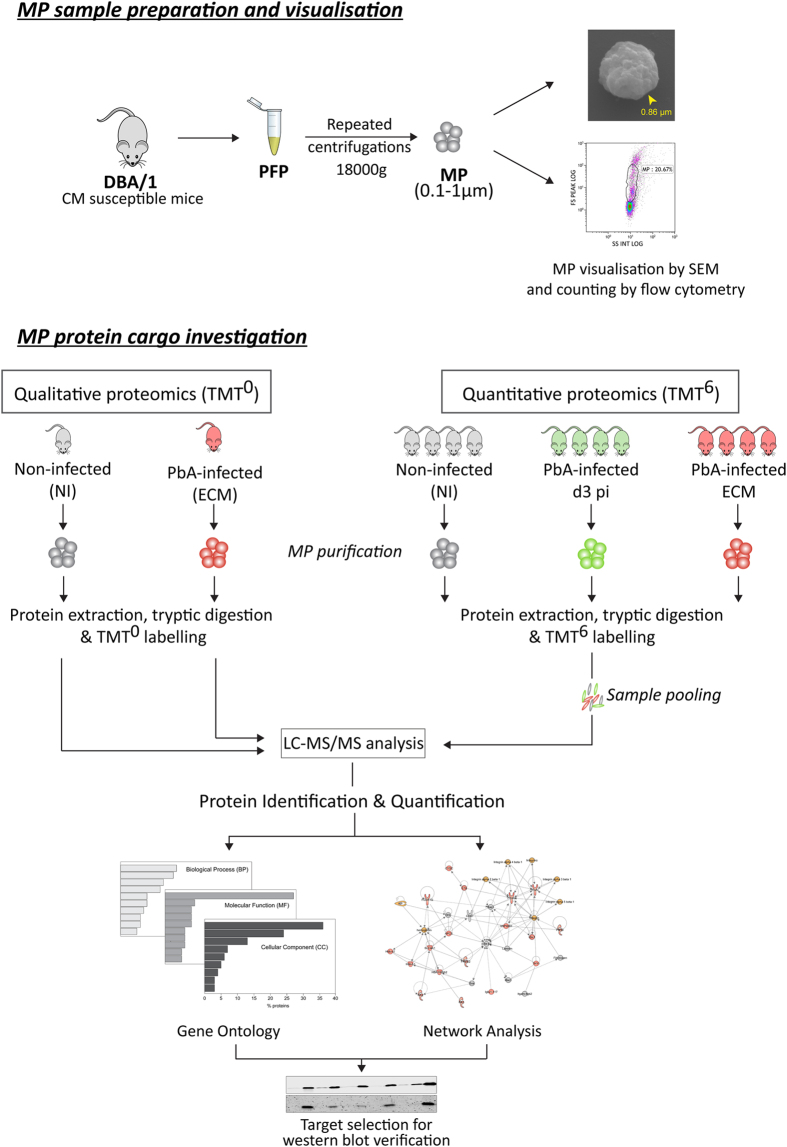
Experimental design. Graphical summary of the experimental design applied in the present study. PFP = platelet free plasma; MP = microparticle; SEM = scanning electron microscopy; NI = non-infected; d3 pi = day 3 post-infection; ECM = experimental cerebral malaria (d8 post-infection). The mouse image was obtained at Pixabay.com.

A graphical representation of the experimental design used in the present study is depicted in Fig. 1.

### MP visualisation by scanning electron microscopy (SEM) and counting by flow cytometry

**Figure 2:**
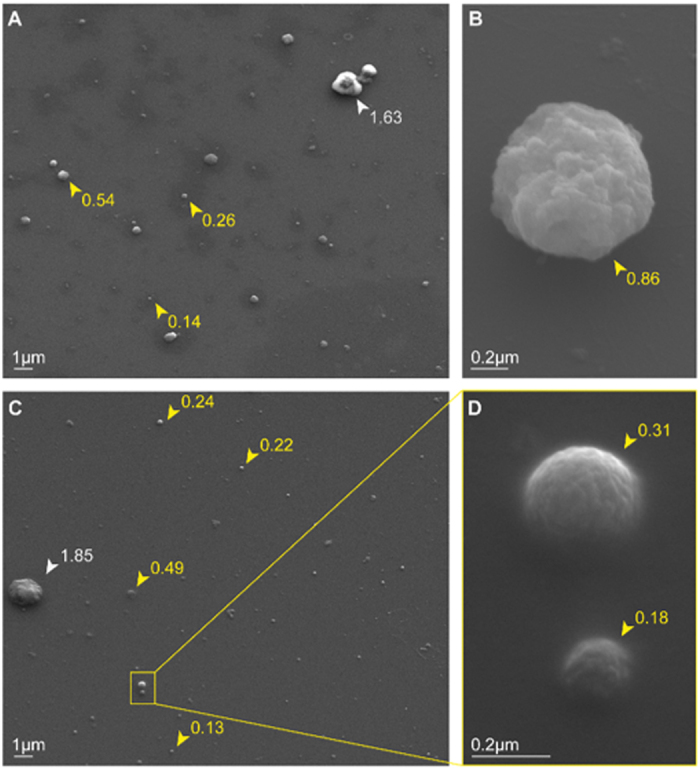
Murine plasma MP visualized by Scanning Electron Microscopy. Plasma MP purified from a non-infected DBA/1 mouse have been imaged with a Zeiss Ultra FESEM. (**A**,**C**) Magnification x4000. The majority of the visualized vesicles have size corresponding to MP (0.1–1 μm - yellow arrowheads), while only one bigger element (white arrowheads) was visualised on each image probably corresponding to small aggregates of MP or microplatelets. (**B**,**D**) Visualisation of plasma MP at a higher magnification, ×23920 and 52160, respectively. Numbers beside arrowheads indicate the measured vesicle diameter expressed in μm.

To demonstrate the efficiency of our purification method, plasma MP from a NI mouse were imaged by SEM (Fig. 2). Figure 2A and C clearly show that the predominant population of purified vesicles has a size range compatible to that of MP (0.1–1 μm); only a minimal contamination of larger elements, which might correspond to cell debris or platelets, was visualised.

When counted by flow cytometry in PFP samples, the number of Annexin V^+^ MP/μL in ECM PFP was 2.5 and 2.1 times higher than in NI and d3 pi PFP, respectively (Supplementary Figure S1).

### Proteomics analyses

#### TMT^0^ qualitative experiment

**Figure 3:**
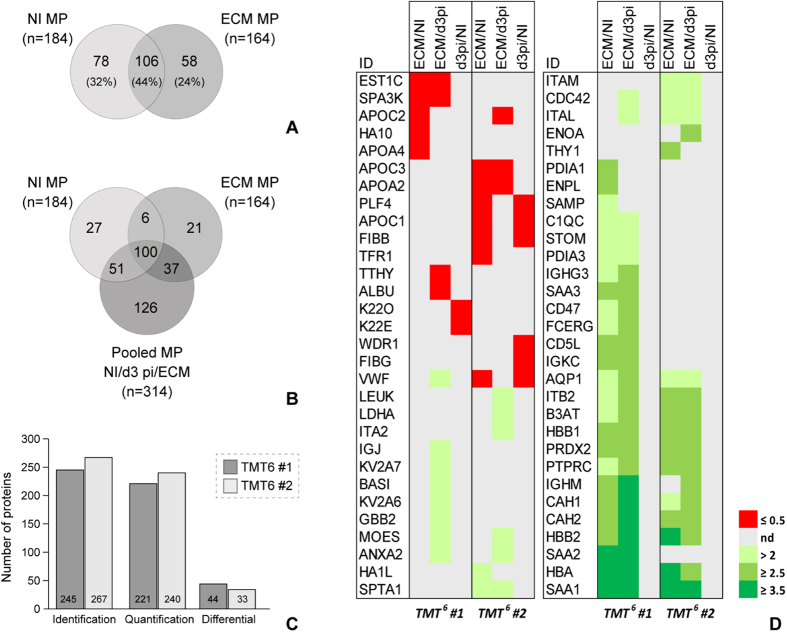
Identification and quantification results. (**A**) Proteins identified in the plasma MP from a non-infected mouse (NI) and a PbA-infected mouse at the stage of experimental cerebral malaria (ECM) (TMT^0^ experiment). The two samples shared 44% of the identifications. Only proteins identified with minimum 2 peptides and FDR ≤1% have been considered. (**B**) Comparison of the proteins identified in the pooled NI – d3 pi – ECM samples (TMT^6^#1 and TMT^6^#2 experiments) and those identified in the individual NI and ECM samples (TMT^0^ experiment). Globally, 368 murine plasma microparticle proteins have been identified. (**C**) Quantitative results obtained from the two TMT^6^ experiments. Proteins differentially abundant in MP were defined as having a p-value < 0.001 and a ratio ≥2.1 or ≤0.5. (**D**) Heat map showing the level of expression of the proteins found to be significantly differentially abundant for the three computed ratios, i.e. ECM/d3 pi, ECM/NI and d3 pi/NI, in the two quantitative experiments. *nd* = *non differential proteins*.

In the TMT^0^ experiment we identified 184 NI MP proteins and 164 ECM MP proteins (2 unique peptides, FDR ≤1%, Fig. 3A).

The complete lists of identified proteins together with the identification details are reported in Supplementary MS Information-Identification. The percentage of TMT tagging – calculated as the proportion of peptides having a TMT^0^ at the N-terminus within the total identified peptides – was ≥97% in both samples, demonstrating the feasibility of the approach.

The comparison of the identified proteins between the two samples showed that 44% of the identifications were shared, 32% were only identified in the NI sample and 24% only in the ECM sample.

#### TMT^6^ quantitative experiments

In the two TMT^6^ quantitative experiments (TMT^6^-1 and TMT^6^-2), 314 proteins were overall identified with at least 2 unique peptides and FDR ≤1%. The good technical efficiency of the TMT^6^ experiments was evaluated through the percentage of labelled peptides (≥94%) and the distribution, across the 6 tags, of the mean relative intensity obtained for the spiked bovine beta-lactoglobulin (coefficient of variation – CV <20%).

Considering the two TMT^0^ and the two TMT^6^ experiments together, we globally identified 368 murine plasma MP proteins (2 unique peptides, FDR ≤1%) (Fig. 3B).

Quantitative information was obtained for 221 and 240 proteins in the TMT^6^-1 and TMT^6^-2 experiments, respectively and, of these, 67% were commonly quantified in the two experiments. Among all quantified proteins (TMT^6^-1 + TMT^6^-2), 60 were significantly different in abundance (Mann-Whitney *U* test, p ≤ 0.001) according to IsoQuant statistics and the calculated ratio threshold of 2.1 for increased proteins and 0.5 for decreased proteins (Fig. 3C,D and Supplementary MS information-Quantification). When the ECM/d3 pi ratio was considered, 38 proteins had significantly increased and 7 significantly decreased abundance. Thirty-one proteins were increased in ECM compared to NI MP, 26 of which had also higher abundance in ECM compared to d3 pi, and 12 proteins had an ECM/NI ratio ≤0.5. Finally, for the comparison d3 pi/NI, 8 proteins were significantly decreased and none were significantly increased, indicating that this is likely too early a time point in the infection to detect differences in MP cargo. This is in agreement with previous results on the number and phenotype of circulating MP in ECM^18^. The complete list of proteins whose abundance in MP was significantly affected during the infection is reported in Fig. 3D.

When *Plasmodium berghei* proteins were searched, two proteins - intra-erythrocytic *P*. *berghei*-induced structures protein 1 and merozoite surface protein-1 - were detected (2 unique peptides, FDR ≤1%) in the TMT^0^ ECM sample, after exclusion of all peptides also matching murine proteins (Supplementary MS information-Identification). In all other samples, no parasite protein could be identified with sufficient confidence.

The mass spectrometry data have been deposited to the ProteomeXchange Consortium (http://proteome central.proteomexchange.org)^22^ via the PRIDE partner repository^23^ with the dataset identifier PXD003772.

### Gene Ontology analysis

**Figure 4:**
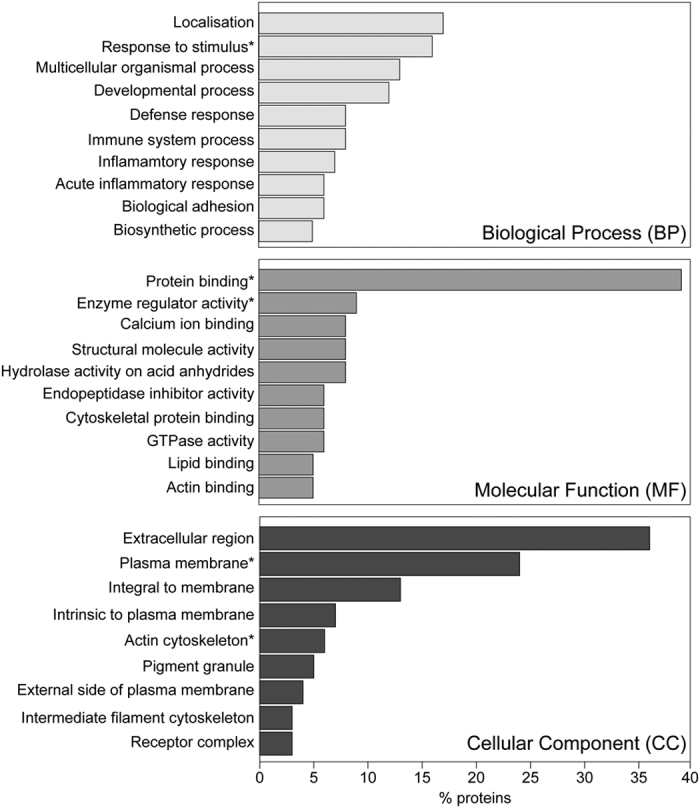
Gene Ontology analysis. Top-10 GO terms represented across all 368 identified murine plasma MP proteins. (**A**) Biological Process GO terms; (**B**) Molecular Function GO terms; (**C**) Cellular Component GO terms. For each category only significantly represented GO terms have been considered (adjusted p-value < 0.0001) and the % of proteins belonging to each term is reported. *Indicates potentially relevant GO terms in the context of MP and ECM.

All murine plasma MP proteins identified in our proteomics experiments (n = 368) were subjected to Gene Ontology (GO) analysis. For each GO category (i.e., biological process – BP, molecular function – MF and cellular component – CC), the top-10 most represented GO terms are reported in Fig. 4.

As shown, the majority of plasma MP proteins were involved in localisation processes and in response to stimulus, with the immune and inflammatory responses appearing among the top 10 BP, probably as a contribution of proteins whose expression was induced by the infection. Considering the CC terms, the identified proteins were highly significantly associated with the cytoskeleton, the plasma membrane and vesicles. Finally, the MF GO terms showed that the proteins identified from murine plasma MP have important active roles, being highly significantly associated with binding and regulatory activities.

### Network and upstream regulator analyses

To highlight proteins associated with the ECM pathological state, a list comprising all those significantly overabundant in ECM MP compared either to d3 pi or to NI (n = 42) and all proteins uniquely identified in the TMT^0^ ECM MP sample (n = 21) was created (ECM-associated proteins, Table 1). Similarly, a list of NI-associated proteins (n = 43) comprised 16 proteins significantly overabundant in NI MP, compared either to d3 pi or to ECM MP, and 27 only identified in NI MP (Supplementary Table 1).

**Figure 5:**
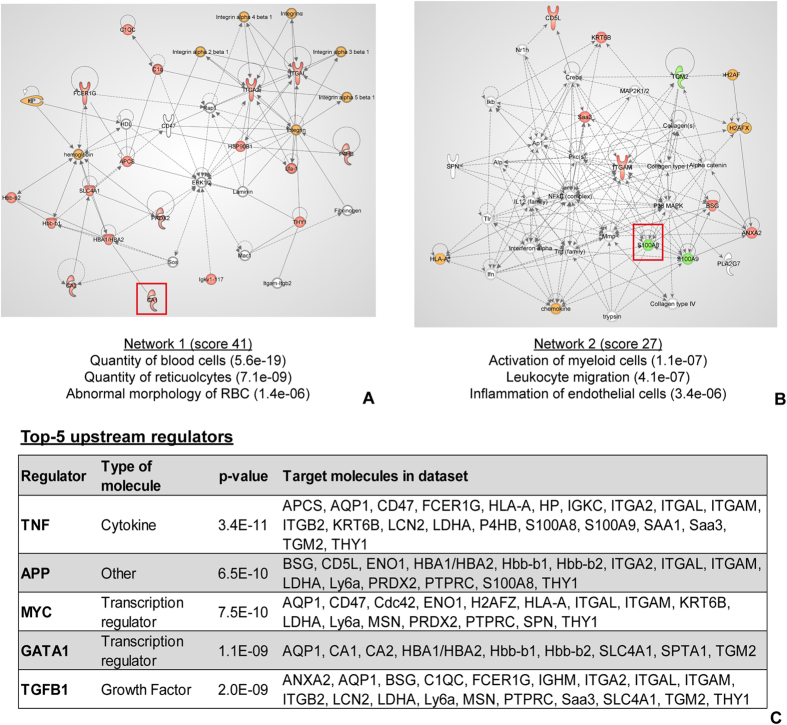
Network and upstream analyses of ECM-associated proteins. (**A**,**B**) Most relevant networks showing the connectivity between MP proteins experimentally identified as ECM-associated (n = 63). Red: proteins identified as significantly over-expressed in ECM; Green: proteins uniquely identified in the ECM sample; Orange: multimeric proteins for which one or more chains have been identified as over-expressed in ECM. The most important biological functions, associated with each network, in the context of ECM, are reported at the bottom of the network. Protein targets selected for verification, CA-I and S100A8, are highlighted by a red square. (**C**) Top-5 molecules that are highly significantly likely to regulate the proteins experimentally found to be ECM-associated. For each regulator the list of target proteins in the experimental dataset is given. *p-value*: Fisher’s exact test, indication of the overlap between the proteins in the dataset and the genes known to be affected by the regulator.

The 63 ECM-associated proteins were analysed with IPA Ingenuity to highlight significantly represented networks and predicted upstream regulators. The top two molecular networks are reported in Fig. 5A and B and for each the most interesting significant molecular functions were selected. The network with the highest score involved 18 molecules of the experimental dataset and was associated (p < 0.0001) with molecular functions affecting the quantity of blood cells and of reticulocytes, and RBC morphology. The second network, involving 13 molecules of the dataset, was significantly associated (p < 0.0001) with the activation of myeloid cells, leukocyte migration and inflammation of endothelial cells.

The upstream regulator analysis predicted, with high significance, that tumour necrosis factor (TNF), amyloid precursor proteins, MYC, erythroid transcription factor (GATA 1) and transforming growth factor beta (TGFβ) were highly likely to regulate the expression of the proteins found to be associated with ECM MP. The detailed results of the upstream regulator analysis are reported in Fig. 5C.

The same analysis was performed on the list of NI-associated proteins (n = 43) and the top two networks were significantly associated with lipid metabolism and with haemostasis (Supplementary Figure S2).

### CA-I and S100A8 verification by western blot

To verify our results, two proteins – carbonic anhydrase 1 (CA-I) and S100A8 - were selected, based on their biological functions and proteomics results, for further evaluation on a larger number of newly collected samples from DBA/1 mice (n = 16). CA-I (identified with 6 peptide spectral matches - PSM, 4 unique peptides and 17.31% sequence coverage) was quantified in both TMT^6^-1 and -2 experiments as overabundant in ECM MP compared to both NI (ECM/NI = 2.9 and 2.4, respectively) and d3 pi MP (ECM/d3 pi = 3.7 and 2.7, respectively). S100A8 was uniquely identified in ECM MP (3 PSM, 3 unique peptides and 54.55% sequence coverage).

**Figure 6:**
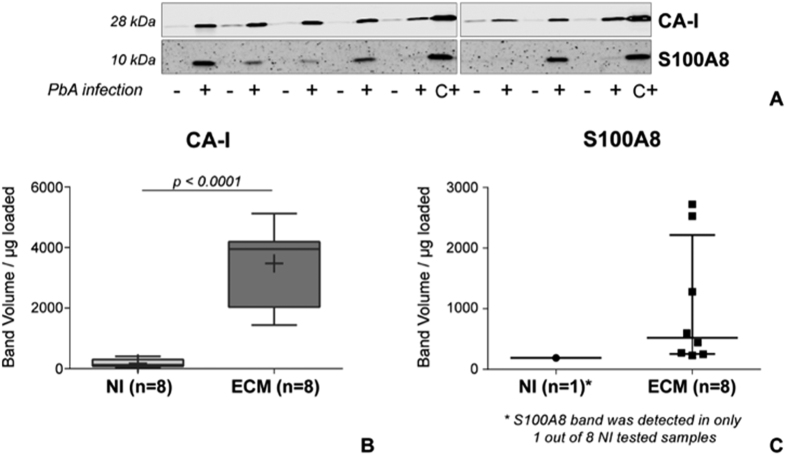
Western blot results for CA-I and S100A8 detection in murine MP. (**A**) Detection of CA-I and S100A8 in MP samples (2.5 μg/lane left blot image, 1.8 μg/lane right blot image) from non-infected (−, NI, n = 8) and PbA-infected (+, ECM, n = 8) DBA/1 mice. A positive control (C+) consisting of murine spleen extract was included in each experiment. (**B**) Quantification of CA-I expression in murine plasma MP, showing its significantly higher expression in ECM samples (t-test). In each boxplot mean is reported as +. (**C**) S100A8 was detectable in only 1 out of 8 NI samples, while it was expressed in all ECM samples. The quantification of the detected bands is reported in the graph, where the horizontal line represents the median band volume and the error bars the interquartile range.

As shown in Fig. 6, CA-I abundance was confirmed to be significantly higher in ECM MP (n = 8) compared to NI samples (n = 8) (t-test, p < 0.0001). A 10 kDa band corresponding to S100A8 protein was detected in all 8 ECM samples but in only 1 out of 8 NI MP, confirming proteomics results where it was only identified in PbA-infected MP. Unfortunately, to the best of our knowledge, a housekeeping protein for plasma MP has not been described yet. When the abundance of γ-actin was assessed on the same samples, a more intense signal was detected in NI samples indicating that actin is not an adequate loading control due to the high variability in protein composition of plasma MP, but also showing that our experiments were not biased towards a higher amount of proteins loaded in ECM samples (Supplementary Figure S3).

S100A8 and CA-I were also assessed in the platelet-free plasma (PFP) and MP-free plasma (MFP) of 10 out of the 16 samples (Supplementary Figure S4). CA-I was expressed in both PFP and MFP but no difference was observed between the 2 types of samples (PFP vs. MFP, Wilcoxon matched-pairs signed rank test) nor between groups (NI vs. ECM, Mann-Whitney *U* test). Interestingly, S100A8 was detected in only 2 out of 5 ECM PFP (and in the corresponding MFP) and in none of the NI samples.

To exclude the possibility of an association between the obtained results and the DBA/1 genotype, CA-I and S100A8 were assessed by WB in MP and PFP samples from C57BL/6 mice (n = 4 NI, n = 4 PbA-infected - ECM) (Supplementary Figure S5). CA-I was significantly more abundant in both MP and PFP samples from ECM mice compared to NI, while S100A8 was only detected in ECM MP, confirming the results obtained by both proteomics and WB in the DBA/1 model. S100A8 was not detected in the PFP of C57BL/6 mice, both NI and ECM.

Finally, the two proteins were also assessed in MP isolated from PFP obtained from children suffering either from CM or from asymptomatic malaria (AM), as well as from healthy community controls (CC) (n = 7 in each group) (Supplementary Figure S6). S100A8 was significantly (one-way ANOVA, p = 0.006) more abundant in MP from CM patients compared to both AM and CC (Dunnet’s multiple comparison post-test, p = 0.0069 and p = 0.0134, respectively). CA-I was not significantly different between the three groups, however its abundance in CM MP was almost double than in AM MP (mean CM/mean AM = 1.9).

## Discussion

Host-derived plasma microparticles (MP) are well recognised as being involved in the pathogenesis of both human and experimental CM^17^,^18^,^20^,^21^, however the contribution of their protein cargo to the pathological mechanisms leading to this syndrome has not been deciphered yet. MP are known to contain proteins derived from the cell of origin, and this cargo doesn’t seem to be the consequence of a casual uptake from the parent cell’s cytoplasm, but rather the result of specific packaging mechanisms^9^,^24^ making these vesicles important players in intercellular communication^11^. To try to achieve new insights into the pathogenesis of CM and to better understand the role of MP in this syndrome, we have established and validated a qualitative and quantitative characterisation of the protein content of plasma MP released during ECM. To our knowledge, this is the first time that this strategy is applied to ECM and that the protein cargo of MP obtained from individual mice is successfully investigated by TMT quantitative proteomics. Only few other studies have used proteomics to investigate extracellular vesicles in malaria models, including *P*. *yoeli*-infected mice or *in vitro* cultured pRBC^25^,^26^.

Through our proteomics investigations we overall identified 368 plasma MP proteins, 290 of which were also quantified. These results are comparable with, or higher than, other proteomics studies on human circulating MP^27^,^28^ and murine plasma exosomes^25^. The list of identified proteins included some of the most abundant plasma proteins, such as albumin, which however were not significantly different between samples. Although a minimal contamination from plasma proteins can never be excluded, some of these proteins have already been reported to be present within MP even after 10 washes of the vesicle pellet or reported as part of the core proteome of human plasma MP^27^,^29^. When the immune-depletion of albumin and immunoglobulins was tested on our samples, the total number of identified proteins increased by 14% (data not shown); despite the increase in the number of identified proteins, such a sample preparation wouldn’t have been compatible with the downstream quantitative analysis of MP proteins derived from individual animals due to the limited amount of proteins available. For these reasons, we instead reduced the contamination of plasma proteins with two washes of the MP pellet, and we increased the number of identified peptides by using a long gradient at the LC-MS/MS level.

To comprehensively evaluate the MP protein cargo, we performed a gene ontology analysis and showed that these proteins reflect both MP mechanism of formation - with a significant representation of cytoskeletal and structural proteins - and their active biological functions, including binding, enzymatic and regulatory activities. This result further sustains that circulating MP released by the host in response to a certain stimulus, transport biologically active molecules that are likely to mediate physiological and pathological processes, as already proposed^24^,^30^,^31^,^32^. Interestingly, a number of proteins already proposed to be associated with parasite virulence and intra-erythrocytic life stage, including basigin, peroxiredoxin 2 and aquaporin 9^33^,^34^,^35^, or associated to the phenomenon of adhesion to the brain microvasculature (integrins) have been identified within ECM MP.

A deeper analysis revealed that these ECM-associated proteins were interconnected in two distinct networks reflecting the pathological state of the sample from which they are derived. The first network involved molecules and pathways significantly associated with RBC number and morphology, indicating that RBC-MP represent an important proportion of circulating vesicles during ECM, as already reported^18^, and that their protein cargo reflects the known alteration of RBC occurring during the infection^36^. However, whether these proteins are specifically packaged into MP to propagate the infection or are just the result of RBC rupture following the maturation of the parasite is still to be determined. These proteomics results were further verified through the validation of the increased abundance in ECM MP of carbonic anhydrase I (CA-I), a protein participating in this network. CA-I, mainly expressed in RBC and in the gastro-intestinal tract^37^, belongs to a group of proteins involved in the maintenance of the acid-base homeostasis of blood and tissues through the reversible catalysis of carbon dioxide and bicarbonate conversion^38^. In severe malaria, the acid-base balance is often affected, as also indicated by the well-reported alteration of the lactate blood levels^39^ and, more recently, by the association of ECM with brain hypoxia and acidosis^40^. Interestingly, CA-I transcript was found to be up-regulated in the plasma of children experiencing severe malaria compared to mild malaria episodes occurring later in the same subjects^41^, further suggesting an interesting role for this protein in CM and raising the possibility that its increased plasma levels are due to MP. In our proteomics analyses we identified and quantified both CA-I and CA-II as significantly over-expressed in ECM MP compared to both NI and d3 pi MP. The muscle-derived CA isoenzyme, CA-III, had been previously identified by affinity proteomics as increased in CM children and as a discriminator between CM and both severe malaria anaemia (SMA) and uncomplicated malaria (UM)^42^.

In our study, CA-I expression was significantly increased in ECM MP compared to NI MP from DBA/1 mice when assessed by WB, however its plasma levels were not different between the two conditions. This might suggest that CA-I could be preferentially packaged in MP - probably derived from RBC - to be transported to other cells to counteract the cellular and tissue acidosis and hypoxia occurring during ECM. These results were confirmed in MP obtained from a different mouse strain (C57BL/6), showing that the increased CA-I expression in ECM MP is not genotype dependent. However, in contrast with what was obtained in DBA/1 mice, when assessed in plasma samples from C57BL/6 mice, CA-I concentration was higher in ECM compared to NI. More in-depth investigations are needed to better understand the contribution of the MP proteome within the plasma proteome.

The second network that was significantly represented among ECM-associated MP proteins comprised molecules implicated in myeloid cell migration, leukocyte activation and inflammatory response in endothelial cells. These functions are known to be important in the pathogenesis of both human and murine CM^4^,^43^,^44^ and, in recent years, particular attention has been paid to the role of the endothelium in CM pathogenesis^45^,^46^,^47^,^48^,^49^. The sequestration of different cell types at the brain microvasculature level - with predominant accumulation of pRBC in human CM and of leukocytes in ECM - is a typical trait of the syndrome^44^,^47^. This adhesion results in the activation of the endothelium and contributes to the endothelial damage associated with CM^46^,^50^. Interestingly, the association of markers of endothelial activation and of platelet adhesion with CM was also shown in patients’ plasma investigated by affinity proteomics^42^. The identification of these proteins further substantiates the active role of MP in ECM: by carrying important molecules between cells, MP could represent primary mediators in cell-to-cell interaction and activation occurring in CM.

Our hypothesis that MP contain proteins actively involved in CM pathogenesis, and in endothelial damage as pointed out by the network analysis, was further confirmed by the upstream regulation analysis, through which two of the most studied molecules in CM pathogenesis, TNF and TGFβ1, were predicted to regulate the MP proteins experimentally detected as ECM-associated. TNF has been reported to be involved, at different levels, in both human and murine CM, including in the interaction between pRBC and leukocytes with the endothelium, sequestration and the release of MP from endothelial cells^20^,^21^,^51^,^52^. Despite the role of TGFβ still being controversial, it has been shown that, on the focal brain obstruction, TGFβ released by adherent and activated platelets contributes to the cerebral endothelium damage^53^.

To further verify our proteomics and prediction analysis results, we assessed the abundance of one of the proteins involved in this second network - S100A8 - that was only identified in ECM-derived MP.

S100A8, also known as myeloid-related protein 8 (MRP-8), is a calcium-binding protein constitutively expressed mainly by neutrophils and monocytes^54^. In human cells and body fluids, it often exists as a heterodimer or tetramer with S100A9 (or MRP14)^55^, which we also identified as uniquely expressed in ECM MP. S100A8/A9 expression can be induced in monocytes, endothelial cells, keratinocytes or epithelial cells by mediators such as LPS and TNF^56^,^57^ and it is often increased in acute and chronic inflammatory conditions^56^, including rheumatoid arthritis, cancers and neurological disorders^58^,^59^ where they seem to play cytokine-like pro-inflammatory effects^55^.

Our verification data confirmed that S100A8 was detected within ECM MP but almost absent in NI MP, in both DBA/1 and C57BL/6 mice. Additionally, its expression was almost not-detectable in plasma samples from both mouse strains, suggesting that MP might be a preferential source of plasma S100A8.

In malaria, elevated serum or plasma levels of S100A8/A9 have been reported in two different studies. Bordmann *et al*. showed that in *P*. *falciparum* malaria-infected children, S100A8/A9 serum concentration correlated with both parasite load and temperature, even though these criteria are no longer sufficient to assess the disease severity^60^. More recently, in PbA-infected BALB/cA mice, an accumulation of S100A8^+^ and S100A9^+^ macrophages in the spleen was shown, as was an increased plasma concentration of the two proteins^61^. In our study, no difference was observed in S100A8 concentration in the spleen between NI and ECM DBA/1 mice (WB, data not shown), however it should be noted that different mouse strains are known to react differently to PbA-infection.

Based on their described biological properties, S100A8/A9 might be interesting players in CM. Indeed, S100A8/A9 are released by circulating neutrophils and monocytes upon contact with the inflamed endothelium^57^ in which they induce the transcription of genes encoding for pro-inflammatory chemokines and adhesion molecules such as ICAM-1^62^. Additionally, S100A8/A9 have been implicated in leukocyte extravasation by increasing the binding capacity of leukocytes to endothelial ICAM-1^62^.

The mechanism of secretion of these proteins in the extracellular environment is still not completely characterised. It has been proposed that their secretion could occur through a tubulin and calcium signalling dependent mechanism^57^,^63^, thus the identification of S100A8 in MP might support these pieces of evidence and represent a new mechanism contributing to S100A8/A9 plasma level.

In our study we focused on the characterisation of host proteins and the alteration of their abundance in MP during ECM infection; however, as reported in other works on the *in vitro* study of pRBC-MP^26^ or on the characterisation of exosomes from *P*. *yoelii*-infected mice^25^, parasite proteins can also be found in extracellular vesicles. Here, we identified only 2 parasite proteins (intra-erythrocytic *P*. *berghei*-induced structures protein 1 and merozoite surface protein-1) in ECM MP analysed using the qualitative approach, potentially as a result of a release of MP from pRBC. This number is lower than what was reported by others, however it should be considered that in order to avoid pooling of samples from multiple mice, our proteomics experiments were performed on a very small amount of protein (approximately 5 μg per sample). Assuming that, similar to exosomes, circulating MP can contain parasite proteins, these would be a minimal proportion of the total protein cargo requiring enrichment and fractionation steps for their detection. Such an investigation should be done in the future to decipher the *in vivo* interaction between the host and the parasite and the role of MP in this. Interestingly, one of the two PbA proteins that we identified within MP was already reported in *P*. *falciparum*-infected red blood cell MP produced *in vitro*^26^.

Our results indicate that the protein content of host-derived plasma MP is severely altered during ECM, compared to the early infection, and that some of the proteins appear to be selectively packaged into these vesicles during the infection. Nonetheless, complementary approaches, such as immune-staining of vesicles released under different infection conditions, should be used to confirm this specific packaging. To further evaluate the implication of MP-associated proteins, and in particular of CA-I and S100A8, in both human and murine CM, additional investigations are needed to establish their exact cellular source and role in disease pathogenesis, using *in vitro* and *in vivo* models. Despite the fact that the two proteins seem to be associated with ECM, as shown by our proteomics results on samples taken at day 3 post-infection, additional investigations should be done using non-CM models to prove the specificity of this association with the severe syndrome. Interestingly, when assessed in MP from human plasma samples from Ugandan children in a pilot study, the two proteins were increased in abundance in MP from children with CM compared to asymptomatic cases and this increase was statistically significant for S100A8. Asymptomatic malaria has recently been pointed out as a debilitating infection that should be considered seriously and treated^64^. These observations suggest that the two proteins might not only be associated to the severe syndrome in mice, but also in humans.

By describing for the first time the protein cargo of murine MP in general and more specifically those released during ECM, and by highlighting new potential molecular players, our results indicate that the cargo of plasma MP is affected during the severe infection and should be considered in the understanding of CM pathogenesis. Additionally, the differences in protein expression between MP and the corresponding PFP indicate that the qualitative and quantitative proteomics investigation of extracellular vesicles has great potential as a complementary strategy to the more common plasma proteomics in biomarker discovery research, since MP are likely to contain molecules specific to the infection or its stage.

The results here presented pave the way to future investigations in multiple directions including the further translation to human clinical samples to try and understand the contribution of plasma MP proteome to malaria severity and to identify new prognostic markers able to predict the short and long term outcome in CM patients.

## Experimental Procedures

### Mice and infection

DBA/1 and C57BL/6 CM-susceptible mice were infected with *Plasmodium berghei* ANKA (PbA) strain and developed a neurological pathology 6–8 days after infection^17^,^65^. Mice were infected and handled as reported elsewhere^18^. Blood was collected retro-orbitally under isoflurane-induced general anaesthesia either at day 3 post-infection (d3 pi) or at the stage of ECM (end-point) in 3.2% sodium citrate. Blood from NI mice was obtained following the same procedure. A total of n = 14 DBA/1 mice were used to perform proteomics experiments; while n = 16 DBA/1 and n = 8 C57BL/6 mice were used to perform verification experiments by WB. The detailed demographic description is reported in Supplementary Figure S1.

#### Ethical approval

All procedures performed in the present study have been approved by the Animal Ethics Committee of the University of Sydney (Projects n: K00/10-2010/3/5317 and K20/6-2011/3/5569) and adhered to the Australian Code of Practice for the Care and Use of Animals for Scientific Purposes.

### Paediatric clinical samples

Platelet free plasma samples was obtained from children enrolled between 2008 and 2012 in Uganda in the context of a study whose details, inclusion and exclusion criteria are reported elsewhere^66^. Written informed consent was obtained from parents or guardians of study participants. The Institutional Review Boards for human studies at Makerere University School of Medicine (Study # 2008-033) and the University of Minnesota granted ethical approval for the study (Study # 0802M27022); all experiments were performed in accordance with relevant guidelines and regulations. The demographic description of the samples used in the present study is reported in Supplementary Figure S6.

### MP preparation, counting by flow cytometry and visualisation by Scanning Electron Microscopy (SEM)

MP were prepared as previously reported^67^. Briefly, whole blood was centrifuged at 1,500 g for 15 min at room temperature to obtain platelet poor plasma (PPP). PPP was then centrifuged twice at 18,000 g for 3 min to remove possible platelet contaminations and/or cell debris and platelet free plasma (PFP) was obtained. MP were finally pelleted at 18,000 g for 45 min at 15 °C. To remove possible plasma protein contaminations (not specifically bound to MP), MP pellet was washed twice with PBS/3.2% sodium citrate and centrifuged at 18,000 g for 45 min after each washing step.

The number of MP in the PFP samples used for proteomics was counted by flow cytometry using Annexin V binding on a Beckman Coulter Gallios^TM^ flow cytometer as previously reported^13^.

To confirm that the isolated population was consistent with plasma MP, vesicles purified from a NI mouse were also imaged by scanning electron microscopy. Samples were prepared as reported by Latham *et al*. and imaged with the Zeiss Ultra FESEM^68^. Imaging was carried out at the Australian Centre for Microscopy & Microanalysis (ACMM), The University of Sydney, Australia.

### TMT^0^ experiment

A pilot experiment using the TMT^0^ approach was performed on MP proteins from one NI and one ECM mouse. For each sample, proteins were extracted from pelleted MP using repeated freeze/thaw cycles^69^, re-suspended in 0.1% Rapigest SF (Waters Corporation) in 0.1 M triethylammonium bicarbonate buffer (TEAB) pH 8.0, alkylated with 50 mM TCEP, reduced with 400 mM iodoacetamide and digested with 0.2 μg/μL trypsin overnight at 37 °C (Promega Corporation). Digested samples were then labelled with the TMT^0^ reagent (Thermo Fisher Scientific) following manufacturer’s instructions, except for the amount of tag used to label the samples. Due to the low amount of proteins in MP samples, one TMT vial (0.8 mg) was used to label two samples. After TMT labelling, samples were cleaned with C18 ultra-micro spin column (Harvard apparatus) and dried under vacuum prior to LC-MS/MS analyses.

### TMT^6^ experiment

MP obtained from 400 μL of PFP from n = 4 NI, n = 4 d3 pi and n = 4 ECM mice were used to perform two parallel TMT^6^ experiments (TMT^6^-1 and TMT^6^-2). Protein extraction, peptide sample preparation and labelling were performed as reported for the TMT^0^. Additionally, each sample was spiked with 0.05 μg of bovine beta-lactoglobulin before protein reduction as an internal control.

Samples analysed in the first TMT^6^ experiment (TMT^6^-1) were labelled as follows: NI with the tags 126 and 127; d3 pi with the tags 128 and 129, and ECM with the tags 130 and 131. In the TMT^6^-2 experiment NI samples were labelled with the tags 128 and 129, d3 pi samples with the tags 130 and 131, and ECM samples with the tags 126 and 127.

After labelling, samples were combined, cleaned with C18 ultra-micro spin column and dried under vacuum prior to LC-MS/MS analyses.

### Tandem mass spectrometry analyses, protein identification and quantification

Tandem mass spectrometry analyses were performed on a QExactive Orbitrap Plus (Thermo Electron) equipped with an Ultimate 3000 HPLC and autosampler system (Dionex). Peptide samples were concentrated and desalted onto a micro C18 pre-column (300 μm × 5 mm, Dionex) that was switched, after 4 min wash, into line with a fritless nano column (75 μm × 15 cm) containing C18 media (1.9 μm, 120 Å, Dr Maisch) manufactured according to Gatlin *et al*.^70^. For the TMT^6^ samples, the analytical peptide separation was run for 260 min at a flow rate of 200 nL/min; while for the TMT^0^ samples, a gradient of 140 min was applied. The detailed description of the MS/MS settings is reported as Supplementary information. Mass spectrometry analyses were carried out at the Bioanalytical Mass Spectrometry Facility, University of New South Wales, Australia.

MS/MS data were analysed using EasyProt platform v2.3^71^. Raw data were converted into.mgf files and peak lists generated with MS convert (ProteoWizard 3.0.7529, http://proteowizard.sourceforge.net/tools.shtml). Protein identifications were obtained by searching peptide spectral matches against the SwissProt/UniProt database (version October 2014 - 546,790 entries) using the following settings: *i*) *Mus musculus* for the taxonomy; *ii*) trypsin as the digestion enzyme with only one missed-cleavage allowed; *iii*) carbamidomethylation of cysteines as fixed modification and oxidation of methionines as variable modification; *iv*) only peptides with at least 6 residues were considered for protein identification and *v*) the precursor ion tolerance was set to 10 ppm. Additionally, the TMT^0^ or the TMT^6^ modifications on lysines and peptide N-termini were added as fixed modifications in the respective experiments. Only proteins identified with at least 2 unique peptides and false discovery rate (FDR) ≤1%, calculated as reported in^71^, were considered as true identification matches.

For the quantitative experiment, protein relative quantification was obtained using IsoQuant embedded in the EasyProt platform. All peptides corresponding to one unique entry in the job, having FDR ≤1% and an intensity signal in each of the 6 TMT tags were considered for quantification.

For each TMT^6^ experiments, protein relative quantification was obtained computing the protein ratios ECM/d3 pi, ECM/NI and d3 pi/NI after isotopic purity correction according to the algorithm given by the manufacturer. To reduce biases due to potential differences in protein amount among the 6 TMT channels, the reporter ion intensities were normalized^71^ prior to ratio calculation and the distribution of the relative intensities of all peptides considered for protein quantification was checked for normality (Shapiro-Wilk normality test). The ratio threshold for considering a protein differentially abundant between the compared conditions was calculated^72^ and the significance level was set at 0.01 (Mann-Whitney *U* test). The technical performance of the two TMT^6^ experiments was evaluated through the rate of labelled peptides and the normalised relative intensity distribution of bovine beta-lactoglobulin across the 6 tags.

For all TMT^0^ and TMT^6^ experiments a second search against the UniProtKB/TrEMBL database (version December 2012 - 4,870,157 entries), choosing the *Plasmodium berghei* taxonomy (ID 5821) was done to search for parasite proteins.

### Gene ontology, network and upstream regulator analyses

To highlight cellular mechanisms, biological processes and molecular functions associated with murine plasma MP proteins, the list of all identified proteins (TMT^0^ and TMT^6^) was submitted to gene ontology (GO) analysis using bioCompendium (http://biocompendium.embl.de/). Only GO terms significantly represented after correction for multiple comparisons were considered (adjusted p-value < 0.0001).

To evaluate the association of the identified MP proteins with CM pathology, two protein sub-datasets were created. A first dataset, called ECM-associated proteins, comprised all proteins found significantly over-abundant in ECM MP compared either to d3 pi or to NI MP in at least one of the two TMT^6^ quantitative experiments, and those exclusively identified in the ECM MP sample (TMT^0^ qualitative experiment). The second dataset, NI-associated proteins, included all proteins significantly increased in NI conditions in the TMT^6^ experiments and those only identified in the NI MP in the TMT^0^ experiment. Both datasets were analysed with Ingenuity^®^ Pathway Analysis - IPA (Ingenuity^®^ System, http://www.ingenuity.com). A network analysis was first performed to evaluate the molecular connectivity and interaction between the proteins in the datasets. This analysis is based on the assumption that highly connected molecules will have a higher influence on the network and that highly interconnected networks will have greater biological significance. Since a network is seen as a set of pathways, the different molecular functions determined to be significantly associated with the network can be highlighted^73^,^74^.

To evaluate ECM- or NI-associated proteins in a broader molecular and biological context, an upstream regulator analysis was also performed listing all genes, RNAs and proteins predicted to regulate the molecules in the experimental dataset.

### Western Blot

#### Murine samples

The expression of two selected candidates, S100A8 and carbonic anhydrase 1 (CA-I), was assessed by WB in MP, PFP and MP-free plasma (MFP) samples.

MP protein concentration was determined after protein extraction using the Qubit protein assay (Life Technologies). For each MP sample, 1 to 2.5 μg of proteins were separated on a polyacrylamide gel and transferred onto a nitrocellulose membrane. For PFP and MFP 2.5 μL of sample were used.

CA-I was detected using a rabbit monoclonal anti-mouse CA-I antibody (0.63 μg/mL, Abcam) and a goat anti-rabbit IgG DyLight^TM^ 800 conjugated secondary antibody (0.15 μg/mL). S100A8 was detected using a goat polyclonal anti-mouse S100A8 antibody (0.4 μg/mL, R&D Systems) and an anti-goat IgG IRDye^®^ 800 conjugated, diluted 1/1000. Monoclonal mAb-2A3 against γ-actin, a kind gift from Christine Chaponnier (University of Geneva), was used at a 0.2 μg/mL and detected with an anti-mouse IgG DyLight 680 conjugated secondary antibody (0.15 μg/mL). The fluorescent signal was detected on an Odyssey Infrared Imaging System (LI-COR Biosciences) and images analysed with ImageQuant TL software (GE Healthcare Life Sciences). Protein expression in MP samples was determined as the measured band volume normalised to the amount of loaded protein.

To establish the appropriate number of mice to be used in WB experiments, a sample size calculation was done based on the variability of the normalised relative intensities of all the peptides used to quantify CA-I in both TMT^6^ experiments, after data logarithmic transformation to obtain a normal distribution. Considering an average standard deviation of 0.3 and 0.7 for ECM and NI samples, respectively, a sample size of n = 8 per group was estimated to be sufficient to detect a significant difference at 0.05 level and a fold-change of 2 with a power of 0.895 (two-sample t test for mean difference with unequal variances).

#### Human samples

MP were prepared from PFP as previously described^13^ and proteins extracted and measured as reported for the murine samples. For each sample, 2 to 3 μg of proteins were separated on 15% polyacrylamide gels. As these samples were obtained from young children the volume of plasma was minimal (160 μL), therefore due to the low amount of proteins obtained from CC MP, pools of two samples age and sex matched were analysed. S100A8 was detected using a rabbit polyclonal primary antibody (2.0 μg/mL, Abcam) and a goat anti-rabbit IgG DyLight^TM^ 800 conjugated secondary antibody (0.15 μg/mL). CA-I was detected with the same antibody used for murine samples.

Statistical analyses were performed using GraphPad Prism 6 (GraphPad Software, Inc.). Parametric or non-parametric statistical tests were applied according to data distribution. Comparisons between two unpaired groups were performed with the t-test or the Mann-Whitney *U* test, while comparisons between three unpaired groups were performed with the one-way ANOVA test followed by the Dunnet’s multiple comparison test. Paired groups were compared using the Wilcoxon matched-pairs signed rank test. All tests were two-tailed and the p-value significance was set at <0.05.

## Additional Information

**How to cite this article**: Tiberti, N. *et al*. Exploring experimental cerebral malaria pathogenesis through the characterisation of host-derived plasma microparticle protein content. *Sci. Rep.* **6**, 37871; doi: 10.1038/srep37871 (2016).

**Publisher's note:** Springer Nature remains neutral with regard to jurisdictional claims in published maps and institutional affiliations.

## Supplementary Material

Supplementary Information

Supplementary Dataset 1

Supplementary Dataset 2

## Figures and Tables

**Table 1:** ECM-associated proteins.

#	AC	ID	Description	ECM/NI Ratio		ECM/d3 pi Ratio	
				TMT^6^ #1	TMT^6^ #2	TMT^6^ #1	TMT^6^ #2
1	P05366	SAA1_MOUSE	Serum amyloid A-1 protein	4.16	3.52	4.66	4.43
2	P05367	SAA2_MOUSE	Amyloid protein A	3.81	—	4.71	—
3	P01942	HBA_MOUSE	Hemoglobin subunit alpha	3.56	3.51	4.16	3.14
4	P02089	HBB2_MOUSE	Hemoglobin subunit beta-2	3.27	3.56	3.70	3.19
5	P00920	CAH2_MOUSE	Carbonic anhydrase 2 (CA-II)	3.39	3.31	4.20	3.20
6	P01872	IGHM_MOUSE	Ig mu chain C region	3.25	—	3.56	2.50
7	Q61171	PRDX2_MOUSE	Peroxiredoxin-2 (TSA)	3.10	3.25	3.29	3.21
8	P02088	HBB1_MOUSE	Hemoglobin subunit beta-1	2.99	3.25	3.32	2.83
9	P08113	ENPL_MOUSE	Endoplasmin (GRP-94) (ERp99)	3.02	—	—	—
10	P04919	B3AT_MOUSE	Band 3 anion transport protein, Isoform Kidney	2.37	3.25	2.83	2.73
11	Q9QWK4	CD5L_MOUSE	CD5 antigen-like	2.75	—	3.22	—
12	P06800	PTPRC_MOUSE	Receptor-type tyrosine-protein phosphatase C, Isoform 3	2.32	3.27	2.85	3.33
13	P09103	PDIA1_MOUSE	Protein disulfide-isomerase (PDI) (ER protein 59)	2.75	—	—	—
14	P01837	IGKC_MOUSE	Ig kappa chain C region	2.64	—	3.41	—
15	P13634	CAH1_MOUSE	Carbonic anhydrase 1 (CA-I)	2.86	2.40	3.67	2.69
16	P04918	SAA3_MOUSE	Serum amyloid A-3 protein	2.52	—	2.67	—
17	P01831	THY1_MOUSE	Thy-1 membrane glycoprotein	—	2.51	—	—
18	P11835	ITB2_MOUSE	Integrin beta-2	2.17	2.70	2.46	2.70
19	Q02013	AQP1_MOUSE	Aquaporin-1 (AQP-1) (DER2)	2.28	2.37	3.38	2.30
20	P27773	PDIA3_MOUSE	Protein disulfide-isomerase A3 (p58) (ERp57) (ERp60)	2.28	—	2.42	—
21	P01897	HA1L_MOUSE	H-2 class I histocompatibility antigen, L-D alpha chain	—	2.28	—	—
22	P24063	ITAL_MOUSE	Integrin alpha-L (LFA-1A) (Ly-15)	—	2.18	2.36	2.23
23	P05555	ITAM_MOUSE	Integrin alpha-M, Isoform 2	—	2.14	—	2.28
24	Q02105	C1QC_MOUSE	Complement C1q subcomponent subunit C	2.13	—	2.25	—
25	P12246	SAMP_MOUSE	Serum amyloid P-component (SAP)	2.12	—	—	—
26	P20491	FCERG_MOUSE	High affinity Ig epsilon receptor subunit gamma	2.12	—	3.15	—
27	P60766	CDC42_MOUSE	Cell division control protein 42 homolog	—	2.12	2.19	2.17
28	P08032	SPTA1_MOUSE	Spectrin alpha chain, erythrocytic 1	—	2.10	—	2.06
29	P03987	IGHG3_MOUSE	Ig gamma-3 chain C region, Isoform 2	2.09	—	2.56	—
30	Q61735	CD47_MOUSE	Leukocyte surface antigen CD47 (IAP)	2.08	—	2.76	—
31	P54116	STOM_MOUSE	Erythrocyte band 7 integral membrane protein	2.08	—	2.30	—
32	P17182	ENOA_MOUSE	Alpha-enolase (NNE)	—	—	—	2.51
33	P07356	ANXA2_MOUSE	Annexin A2 (PAP-IV)	—	—	2.37	2.32
34	P62880	GBB2_MOUSE	Guanine nucleotide-binding protein G(I)/G(S)/G(T) subunit beta-2	—	—	2.28	—
35	Q62469	ITA2_MOUSE	Integrin alpha-2 (GPIa)	—	—	—	2.27
36	P01630	KV2A6_MOUSE	Ig kappa chain V-II region 7S34.1	—	—	2.23	—
37	P26041	MOES_MOUSE	Moesin	—	—	2.30	2.15
38	P18572	BASI_MOUSE	Basigin, Isoform 2	—	—	2.19	—
39	P06151	LDHA_MOUSE	L-lactate dehydrogenase A chain (LDH-A) (LDH-M)	—	—	—	2.19
40	P01631	KV2A7_MOUSE	Ig kappa chain V-II region 26-10	—	—	2.13	—
41	P15702	LEUK_MOUSE	Leukosialin (Ly-48)	—	—	—	2.12
42	P01592	IGJ_MOUSE	Immunoglobulin J chain	—	—	2.07	—
43	Q60963	PAFA_MOUSE	Platelet-activating factor acetylhydrolase (PAF acetylhydrolase)	Only ID			
44	P68254	1433T_MOUSE	14-3-3 protein theta	Only ID			
45	Q61646	HPT_MOUSE	Haptoglobin beta	Only ID			
46	P21981	TGM2_MOUSE	Protein-glutamine gamma-glutamyltransferase 2	Only ID			
47	P17156	HSP72_MOUSE	Heat shock-related 70 kDa protein 2	Only ID			
48	P84244	H33_MOUSE	Histone H3.3	Only ID			
49	P31725	S10A9_MOUSE	Protein S100-A9 (MRP-14) (p14)	Only ID			
51	P27661	H2AX_MOUSE	Histone H2AX (H2a/x)	Only ID			
52	P01670	KV3AI_MOUSE	Ig kappa chain V-III region PC 6684	Only ID			
53	P14426	HA13_MOUSE	H-2 class I histocompatibility antigen, D-K alpha chain (H-2D(K)	Only ID			
54	P0CW03	LY6C2_MOUSE	Lymphocyte antigen 6C2 (Ly-6C2)	Only ID			
55	Q8BFZ3	ACTBL_MOUSE	Beta-actin-like protein 2	Only ID			
56	P01749	HVM05_MOUSE	Ig heavy chain V region 3	Only ID			
57	P01646	KV5AD_MOUSE	Ig kappa chain V-V region HP 123E6	Only ID			
58	P27005	S10A8_MOUSE	Protein S100-A8 (MRP-8)	Only ID			
59	P62806	H4_MOUSE	Histone H4	Only ID			
60	P11672	NGAL_MOUSE	Neutrophil gelatinase-associated lipocalin (NGAL)	Only ID			
61	Q8CGP1	H2B1K_MOUSE	Histone H2B type 1-K	Only ID			
62	P50446	K2C6A_MOUSE	Keratin, type II cytoskeletal 6 A (CK-6A)	Only ID			
63	O08677	KNG1_MOUSE	Kininogen-1 light chain, Isoform 3	Only ID			

List of proteins significantly over-expressed in ECM either compared to d3 pi or to NI (TMT^6^ experiments #1 and #2) and proteins uniquely identified (Only ID) in the ECM sample (TMT^0^ experiment). —: non differential expression.
