# Supplementary material for: Exploring experimental cerebral malaria pathogenesis through the characterisation of host-derived plasma microparticle protein content
Source: Sci Rep. 2016 Dec 5;6:37871. doi: 10.1038/srep37871 (PMC5137300; doi:10.1038/srep37871)
Supplement: Supplementary Information [file srep37871-s1.pdf]

SUPPLEMENTARY INFORMATION: Exploring experimental cerebral malaria pathogenesis through the characterisation of host-derived plasma microparticle protein content

Natalia Tiberti[1,2a], Sharissa L. Latham[1], Stephen Bush[3], Amy Cohen[1], Robert O. Opoka[4], Chandy C. John[5,6], Annette Juillard[1], Georges E. Grau[1,7], Valéry Combes[1, 2a, 7]*

<u>Supplementary information</u>

Detailed description of the mass spectrometry analyses

MS analyses were performed on a QExactive Orbitrap Plus (Thermo Electron) equipped with an Ultimate 3000 HPLC and autosampler system (Dionex). Peptide samples were concentrated and desalted onto a micro C18 pre-column (300µm x 5mm, Dionex) with H2O:CH3CN (98:2, 0.1% TFA) at 15 µL/min. After 4 min wash, the pre-column was switched (Valco 10 port valve, Dionex) into line with a fritless nano column (75 µm x 15 cm) containing C18 media (1.9 µm, 120 Å, Dr Maisch) manufactured according to Gatlin et al. (35). High voltage (2000 V) was applied to a low volume Titanium union (Valco) with the column oven heated to 45°C (Sonation) and the tip positioned ~0.5 cm from the heated capillary (T=300°C) of the mass spectrometer. For the TMT6 sample, the analytical peptide separation was run at a flow rate of 200 nL/min for 260 min using a gradient of H2O/FA 0.1% (solvent A) and CH3CN/FA 0.1% (solvent B) as follows: 0-4 min 2%B, then to 45%B at 246 min and to 80%B at 248 min, and finally 2%B till 260 min. For the TMT0 samples, a gradient of 140 min was applied as follows: 0-4 min 2%B, then to 45%B at 126 min and to 80%B at 127 min, and finally 2%B till 140min, at a flow rate of 200 nL/min.

The QExactive mass spectrometer was operated in the data dependent mode as previously reported (36). A survey scan m/z spectra 350-1750 was acquired in the Orbitrap with 70,000 resolution at m/z 200, and accumulation target value of 1x106 ions and lockmass enabled (m/z 445.12003). Up to the 10 most abundant ions (>80,000 counts, underfill ratio 10%) with charge state ≥ +2 and <+7 were sequentially isolated (width m/z 2.5) and fragmented in the HCD collision cell (NCE = 30) with a maximum injection time of 100ms, 17,500 resolution at m/z 200 and target value set at 1x105. The lower mass of the scanned range was set at m/z 100 to include TMT reporters.

All mass spectrometry analyses were carried out at the Bioanalytical Mass Spectrometry Facility, University of New South Wales, Australia.

Figure S1. Demographic characteristics of the mice used in the present study. A) Demography of the DBA/1 mice used for the proteomics experiments, both qualitative (TMT^0-NI and TMT^0-ECM) and quantitative (TMT^6-1 and TMT^6-2); **B)** Demography and survival curve of DBA/1 and C57BL/6 mice used for the verification of S100A8 and CA-I in MP by western blot.

Proteomics experiments	**DBA/1**		
	**NI (n=5)**	**d3 pi (n=5)**	**ECM (n=5)**
Age, weeks (Mean ± SD)	14.5 ± 3.9	12.5 ± 4.2	13.8 ± 4.7
Gender, M (n)	5	3	4
Parasitaemia, % (Mean ± SD)*	na	<1	13.9 ± 4.9
MP/µL (Mean ± SEM)	290.9 ± 94.3	347.5 ± 80.6	726.3 ± 313.1

** % parasitaemia measured at the time of blood sampling*

ECM samples were obtained at day8 after infection

A

WB experiments	**DBA/1**		**C57BL/6**	
	**NI (n=8)***	**ECM (n=8)**	**NI (n=4)**	**ECM (n=4)**
Age, weeks (Mean ± SD)†	35 ± 19.2	25.8 ± 9.4	21	21
Gender, F (n)‡	4	4	4	4
Parasitaemia, % (Mean ± SD)‖	na	15.9 ± 3.9	na	8.6 ± 1.2

** Missing information on age and gender for one mouse*

† Mann-Whitney U test, non significant

‡ Fisher's exact test, non significant

‖% parasitaemia measured at the time of blood sampling

ECM samples were obtained at day 6 after infection

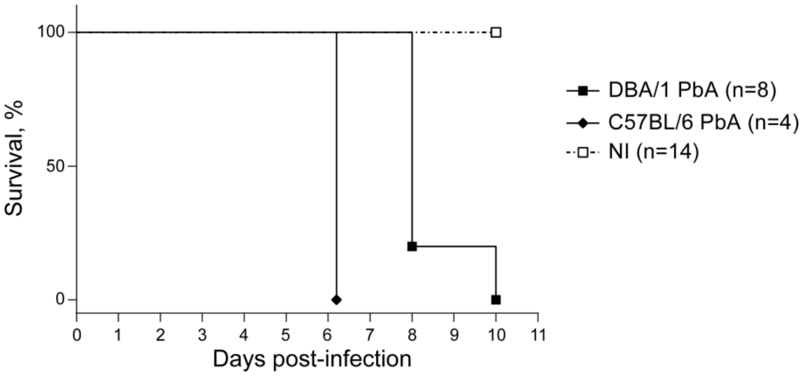

B

Table S1. NI associated proteins. List of proteins (n=43) significantly over-expressed in NI compared either to d3 pi or to ECM (TMT^6 experiments) and proteins only identified in the NI sample (TMT^0 experiment)

				ECM/NI Ratio		d3pi/NI Ratio	
**#**	**AC**	**ID**	**Description**	**TMT^6 #1**	**TMT^6 #2**	**TMT^6 #1**	**TMT^6 #2**
1	Q9Z126	PLF4_MOUSE	Platelet factor 4 (PF-4)	--	0.24	--	0.28
2	P33622	APOC3_MOUSE	Apolipoprotein C-III (Apo-CIII) (ApoC-III)	--	0.40	--	--
3	Q62351	TFR1_MOUSE	Transferrin receptor protein 1 (TR) (TfR) (TfR1) (Trfr)	--	0.40	--	--
4	P34928	APOC1_MOUSE	Truncated apolipoprotein C-I (Apo-CIB') (ApoC-IB')	--	0.41	--	0.50
5	P23953	EST1C_MOUSE	Carboxylesterase 1C	0.43	--	--	--
6	Q8CIZ8	VWF_MOUSE	von Willebrand antigen 2	--	0.45	--	0.34
7	P09813	APOA2_MOUSE	Proapolipoprotein A-II (ProapoA-II)	--	0.47	--	--
8	P01898	HA10_MOUSE	H-2 class I histocompatibility antigen, Q10 alpha chain	0.49		--	--
9	Q05020	APOC2_MOUSE	Apolipoprotein C-II (Apo-CII) (ApoC-II)	0.52	--	--	--
10	Q8K0E8	FIBB_MOUSE	Fibrinogen beta chain [CHAIN 0]	--	0.54	--	0.50
11	P06728	APOA4_MOUSE	Apolipoprotein A-IV (Apo-AIV) (ApoA-IV)	0.54	--	--	--
12	P07759	SPA3K_MOUSE	Serine protease inhibitor A3K (Serpin A3K)	0.54	--	--	--
13	Q3UV17	K22O_MOUSE	Keratin, type II cytoskeletal 2 oral (K76)	--	--	0.52	--
14	Q8VCM7	FIBG_MOUSE	Fibrinogen gamma chain	--	--	--	0.52
15	Q3TTY5	K22E_MOUSE	Keratin, type II cytoskeletal 2 epidermal (CK-2e) (K2e)	--	--	0.54	--
16	O88342	WDR1_MOUSE	WD repeat-containing protein 1 (AIP1)	--	--	--	0.54
17	Q06194	FA8_MOUSE	Coagulation factor VIII	Only ID			
18	P32037	GTR3_MOUSE	Solute carrier family 2, facilitated glucose transporter member 3 (GLUT-3)	Only ID			
19	Q61207	SAP_MOUSE	Sulfated glycoprotein 1 (SGP-1)	Only ID			
20	O88947	FA10_MOUSE	Activated factor Xa heavy chain	Only ID			
21	Q6IRU2	TPM4_MOUSE	Tropomyosin alpha-4 chain	Only ID			
22	P49182	HEP2_MOUSE	Heparin cofactor 2 (HC-II)	Only ID			
23	P47738	ALDH2_MOUSE	Aldehyde dehydrogenase, mitochondrial	Only ID			
24	P70274	SEPP1_MOUSE	Selenoprotein P (SeP)	Only ID			
25	Q91V41	RAB14_MOUSE	Ras-related protein Rab-14	Only ID			
26	Q8K1I3	SPP24_MOUSE	Secreted phosphoprotein 24 (Spp-24)	Only ID			
27	P46638	RB11B_MOUSE	Ras-related protein Rab-11B	Only ID			
28	P39876	TIMP3_MOUSE	Metalloproteinase inhibitor 3 (TIMP-3)	Only ID			
29	Q08879	FBLN1_MOUSE	Fibulin-1 (FIBL-1) (BM-90)	Only ID			

#	AC	ID	Description	Ratio
30	Q8K0D2_ISOFORM_2	HABP2_MOUSE	Hyaluronan-binding protein 2 27 kDa light chain alternate form [ISOFORM 2]	Only ID
31	P33587	PROC_MOUSE	Activation peptide	Only ID
32	P01663	KV3AB_MOUSE	Ig kappa chain V-III region PC 4050	Only ID
33	Q62009_ISOFORM_5	POSTN_MOUSE	Periostin (PN) (OSF-2) [ISOFORM 5]	Only ID
34	P68373	TBA1C_MOUSE	Tubulin alpha-1C chain	Only ID
35	P63001	RAC1_MOUSE	Ras-related C3 botulinum toxin substrate 1	Only ID
36	P52480_ISOFORM_M1	KPYM_MOUSE	Pyruvate kinase PKM [ISOFORM M1]	Only ID
37	A2AQ07	TBB1_MOUSE	Tubulin beta-1 chain	Only ID
38	P35441	TSP1_MOUSE	Thrombospondin-1	Only ID
39	Q8CFG8	CS1B_MOUSE	Complement C1s-B subcomponent light chain	Only ID
40	P45700	MA1A1_MOUSE	Mannosyl-oligosaccharide 1,2-alpha-mannosidase IA	Only ID
41	P97873	LOXL1_MOUSE	Lysyl oxidase homolog 1 [CHAIN 0]	Only ID
42	O55042_ISOFORM_2	SYUA_MOUSE	Alpha-synuclein (NACP) [ISOFORM 2]	Only ID
43	P13020	GELS_MOUSE	Gelsolin (ADF)	Only ID

Figure S2. Network and upstream analysis of NI-associated proteins. **A, B)** Most relevant networks showing the connectivity between proteins experimentally identified as significantly decreased in ECM and/or d3 pi compared to NI samples or only identified in NI mouse sample. The most important biological functions, associated to each network, are reported at the bottom of the network. Yellow: proteins identified as significantly over-expressed in NI; Blue: proteins uniquely identified in the NI sample; Orange: complex molecules for which one or more protein chains/protein components have been identified. **C)** Top-5 molecules significantly likely to regulate the proteins experimentally found to be NI-associated. For each regulator the list of target proteins in the experimental dataset is given. *P-value*: Fisher's exact test, indication of the overlap between the proteins in the dataset and the genes known to be affected by the regulator.

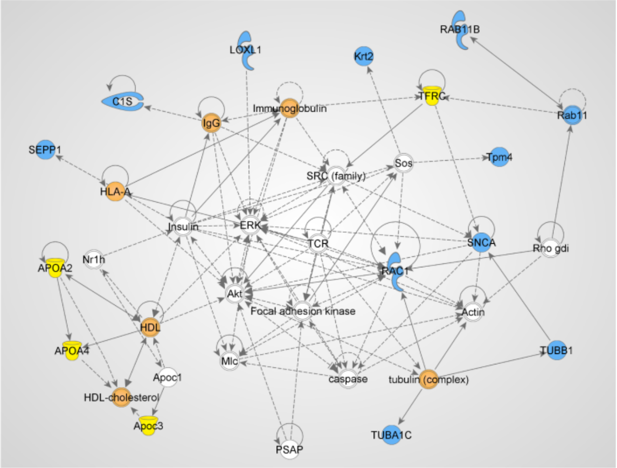

<u>Network 1 (score 38)</u>
Transport of molecules (5.6e-07)
Metabolism of lipid (1.3e-05)
Transport of lipid (1.8e-05)

A

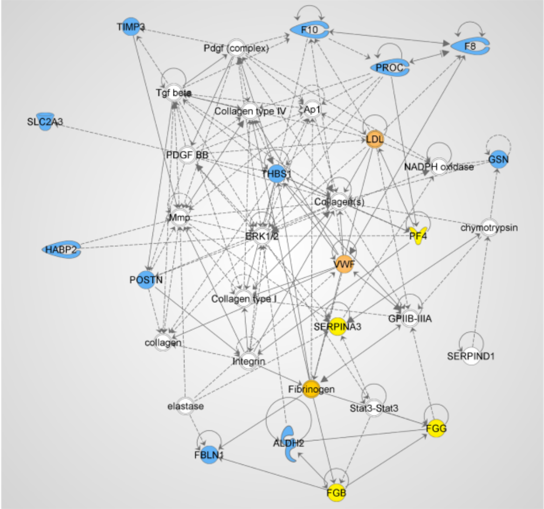

<u>Network 2 (score 38)</u>
Hemostasis (1.0e-09)
Development of vasculature (5.3e-05)
Quantity of blood platelets (1.2e-04)

B

<u>Top-5 up-stream regulators</u>

Regulator	Type of molecule	p-value	Target molecules in dataset
**VIPAS39**	Other	1.0E-07	PF4, THBS1, VWF
**NBEAL2**	Other	1.5E-06	PF4, THBS1, VWF
**TGFB1**	Growth factor	2.8E-06	ALDH2, C1S, GSN, LOXL1, POSTN, RAC1, SERPINA3, SLC2A3, TFRC, THBS1, TIMP3, Tpm4, VWF
**PPARA**	Ligand-dependent	7.5E-06	ALDH2, APOA2, APOA4, Apoc3, C1S, FGB, FGG
**Stat3-Stat3**	Complex	1.0E-05	FGB, FGG, SERPINA3

C

Figure S3. **Detection of γ-actin in MP from DBA/1 mice**. The abundance of γ-actin in MP from NI and ECM DBA/1 mice assessed for CA-I and S100A8 was measured by western blot. As shown, actin abundance in the different samples is highly variable and cannot be considered as a loading control. Nonetheless, the over-abundance of CA-I in ECM MP is confirmed even after actin normalisation, as well as the absence of S100A8 in NI samples.

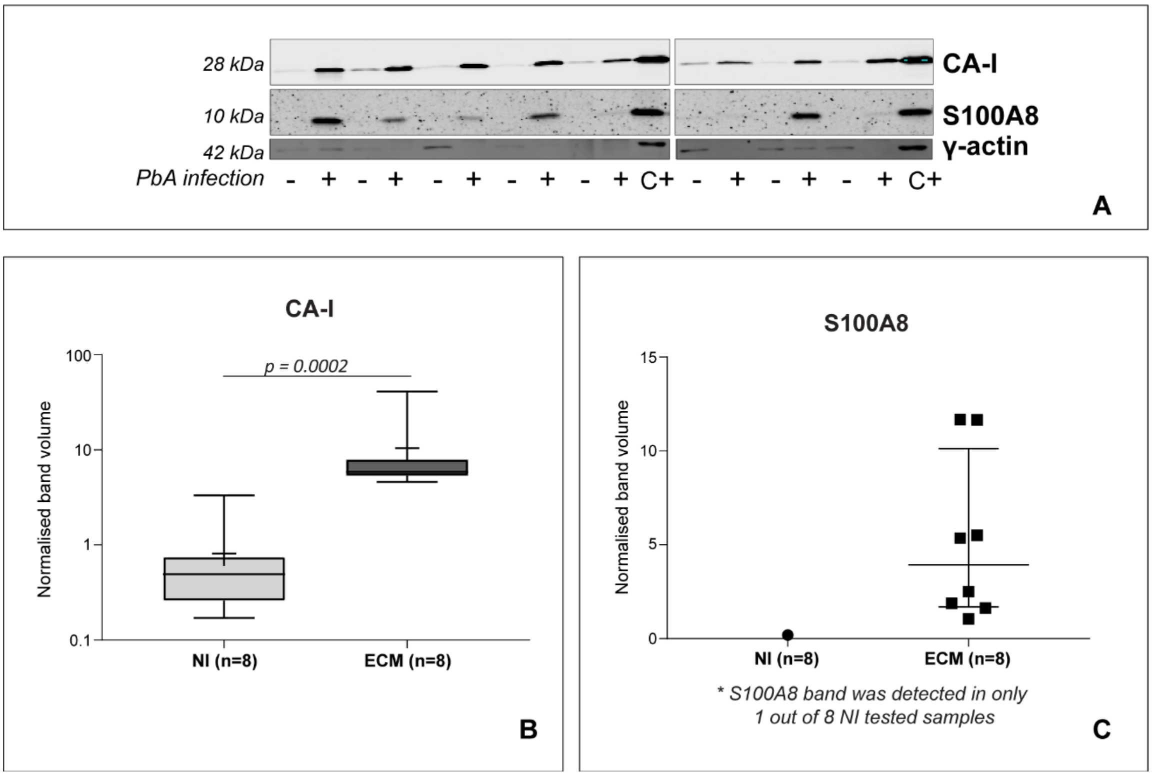

Figure S4. CA-I and S100A8 expression in DBA/1 PFP and MFP. The expression level of CA-I and S100A8 in DBA/1 mice platelet free plasma (PFP) and microparticle free plasma (MFP) was assessed by western blot in n=5 NI mice (-) and n=5 PbA-infected ECM mice (+). C+=positive control, murine spleen.

The quantification of CA-I band volume is reported in the boxplot, where "+" represents the group mean. PFP and MFP groups were compared with the Wilcoxon matched-pairs signed rank test, while ECM and NI samples were compared with the Mann-Whitney *U* test. All comparisons were non-significant. Due to the lack of signal in the majority of the samples, no quantification was done for S100A8.

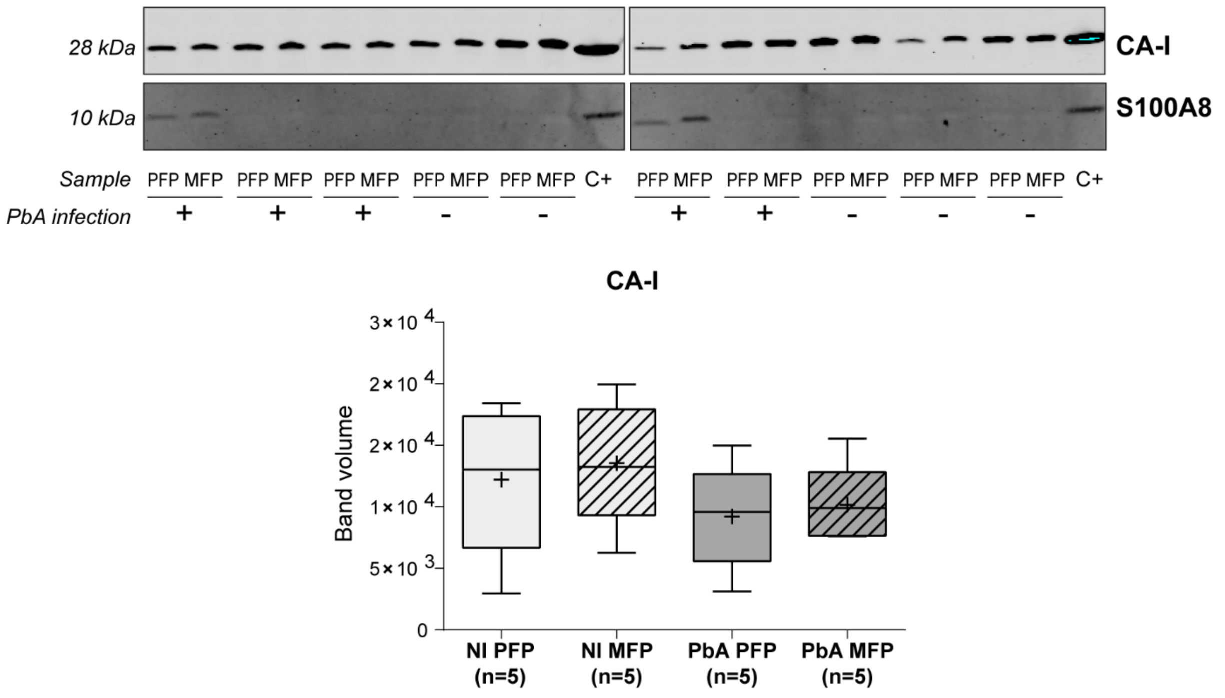

Figure S5. CA-I and S100A8 expression in C57BL/6 mice MP and PFP. The expression level of S100A8 and CA-I in C57BL/6 mice MP and platelet free plasma (PFP) was assessed by western blot in n=4 NI mice and n=4 PbA-infected ECM mice. C+=positive control, murine spleen.

A) Western blot results obtained on MP samples and **B)** the corresponding quantification; for S100A8 no signal was detected in NI samples. **C)** Western blot results obtained on PFP samples and **D)** the corresponding signal quantification for CA-I. Since no signal was detected for S100A8, no quantification was done. Statistical comparisons were done with the t-test.

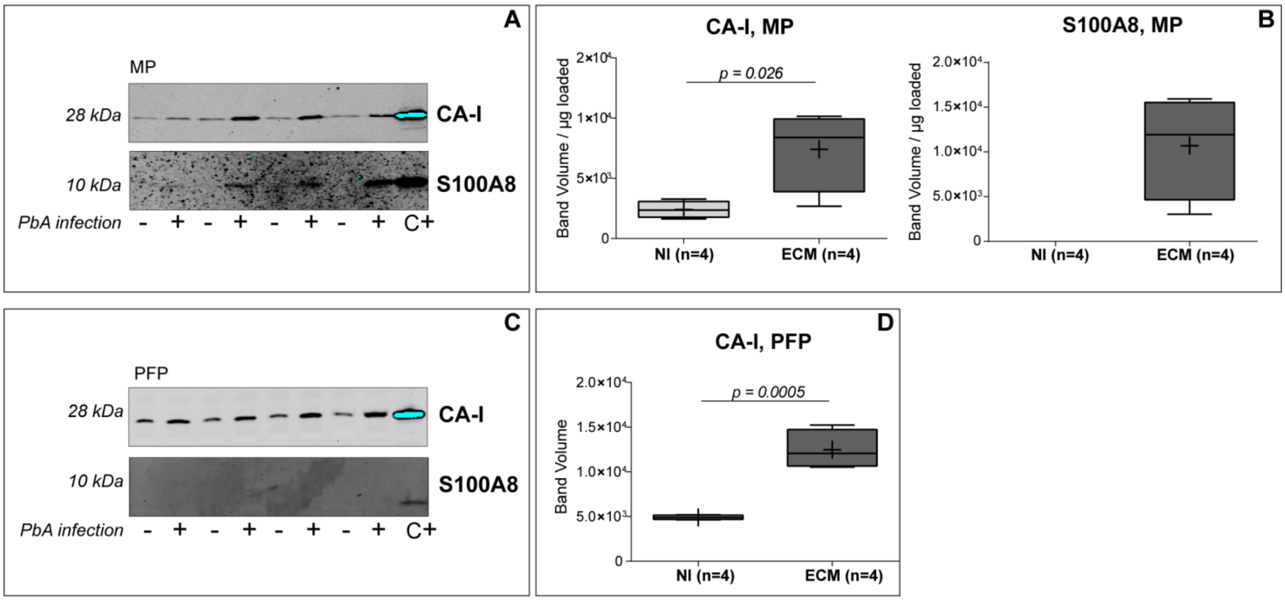

Figure S6. CA-I and S100A8 abundance in MP from clinical samples. CA-I and S100A8 abundance was also assessed on MP samples from a small number of clinical samples. Community healthy controls (CC), asymptomatic malaria patients (AM) and cerebral malaria patients (CM) were included. In order to have a sufficient amount of proteins for CC, a pool of two samples was prepared for each CC lane. **A)** Demographic description of the patients investigated by western blot. **B)** Example of western blot results obtained on human MP. **C)** Quantification results. No statistical difference was detected for CA-I abundance between the 3 groups (Kruskal-Wallis test), however a 1.9 increased abundance was observed in CM compared to AM (mean CM/mean AM). The abundance of S100A8 was significantly higher in MP from CM patients compared to both CC and AM (One-way ANOVA followed by Dunnet's multiple comparison).

	CC (n=14)*	AM (n=8)**	CM (n=7)	*p-value*	Test
**Gender, F (n)**	8	4	3	ns	Chi-square
**Age, years (Mean ± SD)**	4.2 ± 0.95	3.5 ± 1.87	3.3 ± 0.95	ns	Kruskal-Wallis
**Parasites/µl (Mean [range])**	0	8152.5 [1200 - 42840]	262482.9 [17080 - 609400]	0.0012	Mann-Whitney (AM vs CM)

** Seven pools of 2 patiens each were obtained*

** Two samples were pooled, the remaining 6 were analysed individually*

CC: community control, AM: asymptomatic malaria; CM: cerebral malaria

A

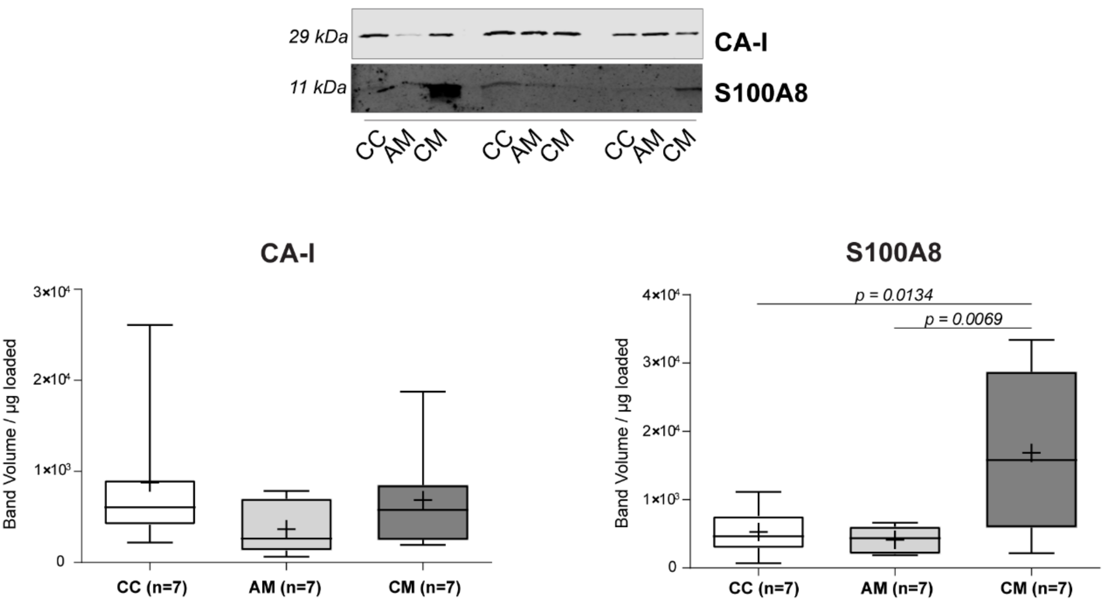

C
